# Teriparatide: an innovative and promising strategy for protecting the blood-spinal cord barrier following spinal cord injury

**DOI:** 10.3389/fphar.2024.1386565

**Published:** 2024-05-06

**Authors:** Moliang Xiong, Yun Feng, Caiguang Luo, Jia Guo, Jihuan Zeng, Liang Deng, Qiang Xiao

**Affiliations:** Department of Orthopedics, Jiangxi Provincial People’s Hospital, The First Affiliated Hospital of Nanchang Medical College, Nanchang, China

**Keywords:** spinal cord injury, blood spinal cord barrier, teriparatide, MMP9, neural damage

## Abstract

The blood-spinal cord barrier (BSCB) is disrupted within minutes of spinal cord injury, leading to increased permeability and secondary spinal cord injury, resulting in more severe neurological damage. The preservation of blood-spinal cord barrier following spinal cord injury plays a crucial role in determining the prognosis. Teriparatide, widely used in clinical treatment for osteoporosis and promoting fracture healing, has been found in our previous study to have the effect of inhibiting the expression of MMP9 and alleviating blood-brain barrier disruption after ischemic stroke, thereby improving neurological damage symptoms. However, there are limited research on whether it has the potential to improve the prognosis of spinal cord injury. This article summarizes the main pathological mechanisms of blood-spinal cord barrier disruption after spinal cord injury and its relationship with Teriparatide, and explores the therapeutic potential of Teriparatide in improving the prognosis of spinal cord injury by reducing blood-spinal cord barrier disruption.

## Graphical Abstract

**Figure:**
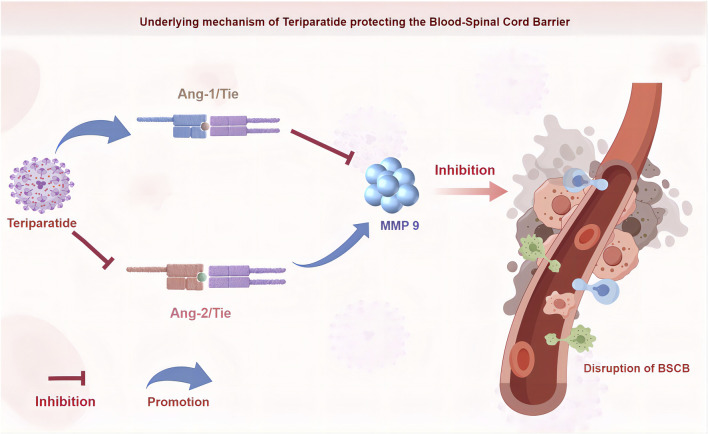
This figure was generated using Figdraw software.

## 1 Introduction

Spinal Cord Injury (SCI) is a devastating neurological disorder caused by mechanical trauma, which has profound physical, emotional, and economic consequences. It often results in impaired motor function, sensory deficits, and autonomic dysfunction, leading to significant impacts on patients, their families, and society as a whole (Badhiwala et al., 2019; Badhiwala et al., 2021; Zipser et al., 2022). Although there have been numerous studies on neuroprotection and regeneration, the treatment options for spinal cord injury patients remain limited (Badhiwala et al., 2021). Research has shown that when the spinal cord is damaged due to various reasons, it can lead to disruption of the blood-spinal cord barrier, resulting in the production of neurotoxic substances that impair the function of synapses and neurons, ultimately leading to permanent neurological deficits (Lee et al., 2012). The blood-spinal cord barrier is an endothelial structure primarily composed of endothelial cells. These cells are interconnected through tight junctions to form a physical barrier between the blood and the spinal cord substance. This barrier prevents toxins, blood cells, and pathogens from entering the spinal cord and maintains a strictly controlled chemical balance in the spinal cord environment, which is necessary for the normal functioning of neural activities (Lee et al., 2012). Given the high incidence and significant harm of spinal cord injury in recent years, prevention of blood-spinal cord barrier disruption has emerged as a potential approach for therapeutic intervention after spinal cord injury, which holds great significance for the prevention and treatment of spinal cord injury.

Teriparatide, also known as recombinant human parathyroid hormone, is an analog of parathyroid hormone composed of the first 34 amino acid residues of the parathyroid hormone molecule. It is the first bone-forming medication approved by the U.S. Food and Drug Administration (FDA) for clinical use. Teriparatide is commonly used in the treatment of postmenopausal osteoporosis and has been shown to significantly increase bone mass in patients with osteoporosis (Langdahl et al., 2017; Edwards et al., 2018; Kendler et al., 2018; Reid and Billington, 2022). Our previous research has found that teriparatide can inhibit the formation of matrix metalloproteinase 9 (MMP9), reduce blood-brain barrier permeability, and improve the prognosis of ischemic stroke. Currently, there is limited literature on the use of teriparatide in the treatment of spinal cord injury. This article aims to review the relevant mechanisms of blood-spinal cord barrier disruption following spinal cord injury, the connection between teriparatide and these mechanisms, and explore the potential therapeutic effects of teriparatide in spinal cord injury.

## 2 The mechanisms underlying disruption of the blood-spinal cord barrier following spinal cord injury

The blood-spinal cord barrier (BSCB) is a fully differentiated and highly specialized endothelial structure within the neurovascular unit (NVU). It primarily consists of endothelial cells, perivascular cells, astrocytes, and the basement membrane, connected by various tight junction proteins and adhesion molecules. It serves as the most important interface mediating substance exchange between blood and the spinal cord, restricting the entry of plasma components and blood cells into the spinal cord. The BSCB maintains the ion homeostasis necessary for neural signal transmission in spinal cord tissue, as well as the separation of central and peripheral neurotransmitters to reduce neural crosstalk. Additionally, the BSCB ensures a low-protein microenvironment within the spinal cord substance, supporting immune surveillance and protecting the spinal cord during trauma and infection (Lee et al., 2012; Jin et al., 2021; Martins et al., 2023). Most previous studies have predominantly posited that the blood-spinal cord barrier is an extension of the blood-brain barrier (BBB), potentially sharing similar morphological structures and functions to maintain homeostasis in the central nervous system (Bartanusz et al., 2011; He et al., 2023). However, recent research has identified certain differences between the blood-brain barrier and the blood-spinal cord barrier, primarily manifested in the ultrastructure of endothelial cells. For instance, glycogen deposits are only present on BSCB microvessels, leading to increased permeability to tracers and cytokines. Additionally, there is a decrease in the expression of transport proteins, tight junction proteins, and adhesion junction proteins (Bartanusz et al., 2011; Jin et al., 2021), Consequently, these differences contribute to the disparity in permeability between the BBB and BSCB. Additionally, studies have shown that the BSCB exhibits higher permeability than the BBB (Bartanusz et al., 2011).

The impairment of blood-spinal cord barrier is associated with various central nervous system disorders. Currently, the most extensively studied condition in this regard is the disruption of blood-spinal cord barrier following spinal cord injury (Bartanusz et al., 2011). SCI can be categorized into the initial damage occurring at the time of injury and a subsequent complex cascade of secondary injuries. The traumatic impact during a spinal cord injury leads to the disruption of the BSCB, allowing peripheral immune substances from the blood vessels to enter the spinal cord. This triggers a cascade of downstream pathophysiological responses, such as oxidative stress, necroptosis, apoptosis, ferroptosis, and neuroinflammation, resulting in the death of neurons and glial cells. This causes changes in the tissue and structural architecture of the spinal cord, including the formation of glial scars and cystic cavities. The presence of glial scars, cystic cavities, and the insufficient capabilities for endogenous myelin regeneration and axonal regrowth imply that the spinal cord has poor intrinsic recovery potential. Consequently, SCI often lead to permanent neurological functional deficits (Ahuja et al., 2017; Venkatesh et al., 2019). Research has revealed that BSCB dysfunction can occur within 5 min after spinal cord injury and can persist in a compromised state up to day 56 post-injury (Maikos and Shreiber, 2007; Cohen et al., 2009; Bartanusz et al., 2011). The study conducted by Zhang et al. (Li et al., 2019) found that after spinal cord injury, there were significant dynamic changes in intramedullary pressure. Within 7 h after injury, there was a rapid increase, followed by a slow increase within 8–38 h. From 39–72 h after injury, a decrease in pressure is observed. They discovered that the early rapid increase may be related to spinal cord bleeding, while edema and disruption of the blood-spinal cord barrier were key factors in the subsequent slow increase. After the disruption of the blood-spinal cord barrier, significant leakage can occur, reaching its peak at 24 h post-injury and showing a second peak on the fifth day (Chang and Cao, 2021). It was found that most of the leakage occurs in the pericellular space, and intercellular pathways are less common, with TJ proteins being the main component connecting BSCB cells (Zhou et al., 2023). The study of He et al. (2023) indicates that spinal cord injury can induce lysosomal damage, leading to disruption of the endothelial cell autophagic flow and dysfunction of the autophagy-lysosomal pathway (ALP). This dysfunction ultimately results in degradation of TJ proteins and breakdown of the blood-spinal cord barrier. TJ proteins mainly include zonula occludens (ZO-1) and occludin. They play a crucial role in the BSCB, functioning as both a “barrier” and a “fence” and regulate the selective permeability of the BSCB (Kumar et al., 2017). Studies have shown that there is no significant decrease in TJ proteins in the early stages of spinal cord injury, specifically at the 1st and 3rd hours. However, a significant reduction can be observed from the 8th to the 24th hour (Zhou et al., 2023). The decrease in TJ expression, along with changes in TJ localization or post-transcriptional modifications, are the main reasons leading to an increased permeability of the BSCB (Deli et al., 2005; Chio et al., 2019; Zhou et al., 2023). Chang and Cao (2021) indicates that spinal cord anterior horn damage often leads to more severe lower limb symptoms and blood-spinal cord barrier (BSCB) disruption. The main reason for this may be that tight junction (TJ) proteins are primarily produced by anterior horn gray matter cells. Following anterior horn damage, the formation of TJ proteins decreases, resulting in more severe BSCB disruption. Cohen et al. (2009) study showed that damage to the spinal cord anterior horn often leads to more severe lower limb symptoms and BSCB damage, possibly because TJ proteins are mainly produced by anterior horn gray matter cells, and after anterior horn damage, TJ protein formation decreases, leading to more severe BSCB damage.

However, Zhou et al. (2023) found in their study that after spinal cord injury, the structures and functions of the paracellular connections are disrupted, and the extensive formation of gaps may be the main cause of blood-spinal cord barrier (BSCB) disruption. They discovered that alleviating pathological hemodynamic changes partially reduced the formation of gaps in the BSCB after SCI. They believe that pathological hemodynamic changes play a crucial role in the rapid and extensive BSCB disruption after SCI, possibly through increased pathological shear and transmural forces exerted on the endothelium. Additionally, the study also found that leukocytes start to move along the blood vessels within minutes after spinal cord injury and reach the core of the lesion in the gray matter of the spinal cord within 4 h. Subsequently, leukocytes can actively induce the formation of gaps in the BSCB. When leukocyte extravasation is inhibited, the formation of gaps in the BSCB is reduced, indicating that the involvement of leukocyte migration is involved in the early stages of BSCB leakage after SCI (Zhou et al., 2023). After BSCB injury, there is an increase in permeability, allowing a large number of immune cells, toxins, blood cells, and pathogens to enter the spinal cord, leading to a series of secondary damages such as spinal cord edema, hemorrhage, oxidative stress, and excessive inflammatory reactions (Jin et al., 2021), These further exacerbate the spinal cord injury and cause more severe neurological dysfunction. Increasing evidence suggests that maintaining the integrity of BSCB can serve as a neuroprotective target for reducing spinal cord injury (Jing et al., 2020), Therefore, preserving the integrity of BSCB is beneficial for the repair and improvement of neurological function following spinal cord injury.

Currently, the therapeutic approaches for the BSCB primarily focus on maintaining its normal function by protecting key cells and structures, such as endothelial cells, tight junctions, astrocytes, and the basement membrane, to alleviate spinal cord injury (Jin et al., 2021). Although the protective mechanisms of many drugs have been elucidated, the complex pathophysiology of BSCB disruption has limited the successful transition of effective drugs from the laboratory to clinical use. Jin et al. (2021) summarized the current therapeutic approaches for BSCB, finding that treatment targets may primarily focus on the substructures of the BSCB, such as using new molecules or drugs to maintain the integrity of endothelial cells and inhibit the low-level expression of TJ proteins, thereby reducing the severity of SCI. Additionally, a few studies have focused on the roles of the endoplasmic reticulum (ER), autophagy, and MMPs in the BSCB. Furthermore, they discovered that with the development of regenerative engineering and biomaterial scaffolds, research has been applied to repair the damaged BSCB using stem cells and biomaterials.

## 3 The relationship between blood-spinal cord barrier disruption and matrix metalloproteinases (MMPs)

Research has demonstrated that proteases, particularly matrix metalloproteinases, serve as the primary mediators of blood-spinal cord barrier (BSCB) disruption following spinal cord injury (Noble et al., 2002; Jang et al., 2011), Matrix metalloproteinases (MMPs) are a group of zinc-dependent endopeptidases that play a role in the degradation of extracellular matrix components such as collagen, fibronectin, and laminin. Their overexpression has been implicated in a range of central nervous system (CNS) pathological processes (Zhao et al., 2006; Jang et al., 2011). Up to date, there are 24 structurally and functionally related members of MMPs within mammalian organisms, with each member possessing distinct functionalities (Vandenbroucke and Libert, 2014). Studies have found that MMP9 plays a pivotal role in the immediate abnormal vascular permeability and inflammation after spinal cord injury (Bo Fang et al., 2013), MMP9 rapidly increases after spinal cord injury, reaching its peak at 24 h, significantly decreasing at 72 h, and becoming undetectable 7 days after the injury. Blocking MMP9 formation within 3 days of spinal cord injury can alleviate vascular permeability and improve motor recovery (Noble et al., 2002).

Under normal conditions, MMP9 is expressed at low levels in microglia, astrocytes, and hippocampal neurons. After spinal cord injury, the expression of MMP9 rapidly increases and reaches its peak in inflammatory cells and endothelial cells (Bi et al., 2020), Linda et al. (Noble et al., 2002) found that the abnormal increase of MMP-9 in inflammatory cells and endothelial cells may jointly impair the blood-spinal cord barrier function by degrading the vascular basement membrane. Li et al. (Bi et al., 2020) demonstrated that the activation of MMP9 can lead to the degradation of type IV collagenase and destruction of the extracellular matrix, resulting in increased permeability of the microvascular basement membrane and damage to the blood-spinal cord barrier. Moreover, studies have also found that MMP9 can disrupt the integrity of the BSCB by degrading tight junction proteins such as ZO-1 (Jing et al., 2020).

However, subsequent studies have also indicated that, apart from MMP9, other members of the MMP family are also associated with the disruption of BSCB, The research conducted by Kumar et al. (2017) demonstrated that MMP8 is involved in the degradation of TJ proteins in endothelial cells, thereby disrupting BSCB. Lee et al. (2014) also found that MMP3 can degrade TJ proteins, causing damage to BSCB. Furthermore, research has also found that MMP2 and MMP12 have the ability to regulate vascular permeability and disrupt the integrity of BSCB (Wells et al., 2003; Yang et al., 2013). Although the aforementioned MMPs are all involved in the breakdown of BSCB, most current research suggests that MMP2/MMP9 are the key enzymes responsible for BSCB disruption following spinal cord injury, as they increase BSCB permeability by degrading the basement membrane cells (Min-Sheng Piao et al., 2014; Łukomska et al., 2020). MMP9 can induce protein degradation associated with blood-spinal cord barrier, while upregulation of MMP2 can lead to the initial opening of blood-brain barrier/blood-spinal cord barrier (Ying et al., 2020). Subsequent studies have found that although both MMP2/MMP9 are strongly induced after spinal cord injury, the expression and activation of MMP9 precede those of MMP2. MMP2 starts to increase around 48 h after spinal cord injury, while MMP9 reaches its peak at 24 h after injury. Therefore, MMP9 mainly participates in the early disruption of BSCB after spinal cord injury (Min-Sheng Piao et al., 2014). The study by Gao et al. (2016) found that selective inhibition of MMP2 expression does not alter neural recovery after spinal cord injury. They observed compensatory increase in MMP-9 activity in MMP2 knockout mice following spinal cord injury. Therefore, there is an increasing number of studies focused on inhibiting MMP9 activity, which is currently considered a therapeutic target for improving blood-spinal cord barrier disruption after spinal cord injury.

The integrity of BSCB is related to multiple pathways mediated by MMPs, Wang et al. (Xin et al., 2021) demonstrated that activation of the TIMP2/MMP signaling pathway can reduce the expression of MMP2 and MMP9, alleviate BSCB disruption, and promote neural function recovery after spinal cord injury (SCI). In addition, studies have found that the mTOR/JMJD3 signaling pathway can also mediate the expression of MMP2 and MMP9, regulating the integrity of BSCB (Park et al., 2023). Lee et al. (2014) found that NF-κB is involved in the expression of MMP-3, As an important central nervous system regulator, recent studies have also found that NF-κB can also mediate the expression of MMP9 and regulate the integrity of BSCB (Xin et al., 2023). TLR4 signaling pathway has also been found to be involved in the protection of BSCB, Researchers have discovered that activation of TLR4 can induce polarization of astrocytes into an inflammatory phenotype (Jang et al., 2011), and astrocytes can contribute to blood-brain barrierinjury through the activation of MMP9 (Jing et al., 2020), After inhibiting the activation of TLR4 signaling pathway, It can reduce the expression of MMP9 in endothelial cells, thereby protecting BSCB function (Zhu et al., 2023).

## 4 The relationship between teriparatide and spinal cord injury

The deficiency of neurological function after spinal cord injury (SCI) is related to the level of parathyroid hormone (PTH) (Mechanick et al., 1997), Studies have found that PTH significantly decreases within 1–2 weeks after SCI (Vaziri et al., 1994; Rouleau et al., 2007), According to a study by del Rivero and Bethea (2016) the average plasma PTH level in the control group of mice was 30.65 pg/mg, while the average plasma PTH level in SCI mice was 20.5 pg/mg at 1week post-injury and 14.25 pg/mg at 4 weeks post-injury, indicating a gradual decline in parathyroid hormone. At present, most of the research on the relationship between parathyroid hormone and SCI focuses on the changes in parathyroid hormone levels after SCI, with few studies on the use of parathyroid hormone therapy for SCI.

Teriparatide is a parathyroid hormone analog that is commonly used in the treatment of postmenopausal osteoporosis. It has been shown to significantly increase bone mass in patients with osteoporosis (Langdahl et al., 2017; Edwards et al., 2018; Kendler et al., 2018; Reid and Billington, 2022). In addition, some scholars have utilized its osteogenic properties to treat bone defects in limbs, mandibles, skulls, and other areas (Cohn Yakubovich et al., 2017; Xie et al., 2017; Zhang et al., 2017; Yukata et al., 2018; Zandi et al., 2019), Moreover, it has also been used for the treatment of nonunion fractures with satisfactory outcomes (DeRogatis et al., 2020; Marin, 2021), As for the application of Teriparatide in spinal cord injury, most studies have focused on changes in bone mass and strength after spinal cord injury (Edwards et al., 2018; Haider et al., 2019), and there are few relevant reports on the treatment of SCI with Teriparatide. Further research is needed to determine whether Teriparatide can improve the prognosis of spinal cord injury. Our previous studies have shown that Teriparatide can promote vascular regeneration around the ischemic stroke area and improve neurological symptoms after stroke. Further investigation revealed that Teriparatide can alleviate neuronal damage and improve neuronal function after stroke by inhibiting oxidative stress and neuroinflammation (Xiong et al., 2022). However, whether it has the same effect and its exact mechanisms after spinal cord injury still need further clarification.

## 5 The relationship between teriparatide and BSCB

In our previous study, we utilized Teriparatide to treat the rat MCAO model and found that Teriparatide could induce the generation of Angiopoietin-1 (Ang-1), thereby reducing the disruption of the blood-brain barrier (Xiong et al., 2022), However, the specific mechanisms underlying this effect have not been investigated. Ang-1 is not only a growth factor involved in central nervous system vascular development, maturation, remodeling, and stability, but also a crucial factor in regulating brain and spinal cord vascular function (Herrera et al., 2010; Durham-Lee et al., 2012). Similar to Ang-1, Ang-2 is a secreted growth factor that exerts downstream signaling effects through binding to Tie receptors. In the central nervous system, a dynamic balance between high levels of Ang-1 and low levels of Ang-2 is essential to maintain barrier integrity. After binding to Tie receptors, Ang-1 can induce phosphorylation to inhibit endothelial cell apoptosis, thereby protecting the integrity of the central nervous system barrier. On the other hand, Ang-2 can competitively bind to Tie receptors with Ang-1, inhibiting phosphorylation and mediating endothelial cell death, leading to increased vascular permeability and disruption of the integrity of the central nervous system barrier (Zhu et al., 2005; Gu et al., 2016). Ang-1 levels immediately decrease after spinal cord injury and remain at a low level at all time points examined, while Ang-2 expression continues to increase, starting on the third day after spinal cord injury and reaching its peak at 21 days. It remains highly expressed even at day 70 (Herrera et al., 2010; Durham-Lee et al., 2012).

Researchers have found that Ang-1 can effectively reduce the level of MMP9 mRNA, thereby inhibiting the expression of MMP9 (Wu et al., 2015), Other studies have also found that Ang-1 can modulate the activity of MMPs, counteract the effects of VEGF, and improve the permeability of the central nervous system barrier (Valable et al., 2005). Furthermore, the study by Hou et al. (Hou et al., 2021) found that the binding of Ang-2 to Tie receptors leads to the expression of MMP2 and MMP9. Therefore, promoting the generation of Ang-1 is more beneficial for reducing the disruption of blood-spinal cord barrier (BSCB) after spinal cord injury. Our previous study found that teriparatide can promote the generation of Ang-1 and reduce the expression of MMP9. However, the mutual relationship between them and the specific mechanism have not been thoroughly investigated. Meanwhile, we also discovered that teriparatide can reduce the expression of Ang-2, but these studies mainly focused on ischemic stroke. There are few reports on whether teriparatide has similar mechanisms after spinal cord injury. Therefore, it is a new treatment option to explore whether teriparatide can alleviate the disruption of blood-spinal cord barrier after spinal cord injury by promoting the generation of Ang-1.

In clinical settings, treatment methods for SCI mainly include pharmacotherapy and surgical approaches. Currently, drugs that have entered clinical trials include methylprednisolone sodium succinate, minocycline, riluzole, and basic fibroblast growth factor (Ahuja et al., 2017). Methylprednisolone sodium succinate, which has immunosuppressive effects, can reduce cytotoxicity, but it may lead to severe consequences such as wound infection, elevated blood sugar, obesity, and ischemic necrosis of the femoral head. Minocycline can protect neural functions by reducing oligodendrocyte apoptosis and lowering local inflammation, but it may cause an increase in liver enzyme levels. Additionally, riluzole can prevent the sustained activation of neuronal voltage-gated sodium channels, thereby preventing cell swelling and death, and reducing excitotoxicity, but it can also lead to elevated liver enzymes (Ahuja et al., 2017; Venkatesh et al., 2019). Teriparatide, a derivative of human parathyroid hormone, is currently widely used in the treatment of osteoporosis, with no studies reporting adverse reactions. Moreover, patients with spinal cord injuries are prone to significant bone loss due to prolonged bed rest after the injury. Teriparatide not only treats spinal cord injuries but also promotes bone growth, which is more beneficial for the prognosis of spinal cord injuries.

## 6 Conclusion

In summary, the disruption of the blood-spinal cord barrier after spinal cord injury is primarily associated with the degradation of basement membrane components, leading to increased permeability and exacerbation of neurological damage following spinal cord injury. Currently, research on teriparatide mainly focuses on improving the prognosis of osteoporosis, and there is limited research on improving the integrity of the blood-spinal cord barrier after spinal cord injury. Further studies are needed to clarify this relationship.
